# Study on SAW Methane Sensor Based on Cryptophane-A Composite Film

**DOI:** 10.3390/mi14020266

**Published:** 2023-01-20

**Authors:** Xinlei Liu, Bin Shen, Leiming Jiang, Haiyang Yang, Chunbo Jin, Tianshun Zhou

**Affiliations:** School of Safety Engineering, Heilongjiang University of Science and Technology, Harbin 150022, China

**Keywords:** SAW, methane detection, Cryptophane-A, composite membrane, performance

## Abstract

Surface Acoustic Wave (SAW) methane-sensing technology is a new way to detect methane at room temperature. However, the material and structure of the sensitive film are the important factors affecting the detection performance of the sensor. In this paper—with a SAW methane sensor using graphene–nickel cavitation—a composite film is proposed, which can work at room temperature. A delay linear dual-channel differential oscillator with center frequency of 204.3 MHz and insertion loss of −5.658 dB was designed; Cryptophane-A material was prepared by the “three-step method”. The composite sensitive film was synthesized by a drop coating method, electrochemical deposition method and electroplating method. The composite film was characterized by SEM. The sensor performance test system and gas sensitivity test system were constructed to determine the response performance of the sensor at concentrations of 0~5% CH_4_. The results showed that the sensor had a good response recovery performance in the test concentration range, and the frequency offset was positively correlated with methane concentration. The 90% average response time and recovery times were 41.2 s and 57 s, respectively. The sensor sensitivity was 809.4 ± 6.93 Hz/(1% CH_4_). This study provides a good theoretical basis for the development of surface acoustic-wave methane sensors.

## 1. Introduction

China is a country with high levels of coal output and consumption. However, the poor storage conditions of coal resources and the lack of effective monitoring and prevention measures have led to frequent coal-mine gas accidents. Therefore, it is necessary to develop an efficient and reliable gas sensor to conduct gas monitoring. About 83–89% of the gas is comprised of methane. Methane is colorless and tasteless, concentrations of more than 5% are prone to explosion, and its stable properties make it difficult to detect at room temperature. Methane sensors in China mainly include the infrared absorption type [1,2,3], catalytic combustion type [4,5], gas–sensitive semiconductor type [6,7], light interference type [8,9] and surface acoustic wave type [10,11].

In recent years, surface acoustic wave (SAW) sensors have been widely employed due to the advantages of their small size, low power consumption, high sensitivity and flexible application [12,13]. The SAW device has no selectivity towards gas, but the sensitive film coated on the surface of SAW has selectivity towards gas. The mass conductivity and elasticity coefficients change, leading to a change in the frequency and phase of the sound surface wave. It is through the change of the measurement frequency that the concentration of the gas to be measured is obtained [14,15]. At present, common gas-sensitive film materials include inorganic substances [16,17,18,19,20], organic polymers [21,22,23] and supramolecular compounds [24,25]. Supramolecular compounds are cage structures with a cavity structure, which can recognize specific guest molecules. Supramolecular compounds are suitable for measuring the gas to be measured at room temperature and become the development trend of gas sensitive materials.

Graphene has a high specific surface area and electrostatic adsorption performance, which can adsorb methane molecules. Its good physical properties and application potential are conducive to the integration of surface acoustic wave devices [26,27,28,29]. In recent years, more and more studies have been conducted using graphene as a sensitive film material for SAW devices. For example, SAW devices for measuring carbon monoxide, carbon dioxide [30], hydrogen [31] and humidity. Guo et al. [32] prepared a humidity sensor with graphene as the sensitive medium on a LiNbO_3_ resonator. The performance of a SAW sensor is better because the graphene surface has a good adsorption capacity of water molecules and mass-loading effect. Nash [33] proved that graphene can affect the amplitude and velocity drift of SAW. This method can produce an economical and practical voltage-controlled speed shifter suitable for wireless sensor applications. Whitehead [34] carried out a theoretical study on the variation of SAW propagation on piezoelectric substrates due to the interaction with carriers in graphene. Preciado’s [35] study also highlighted the potential advantages of combining graphene with SAW devices, but graphene applications for SAW methane sensors were not reported on in this study.

Nickel is often used as a catalyst due to its excellent electrical conductivity and catalytic performance [36]. The combination of nickel and graphene can not only enhance the corrosion resistance and wear resistance of the nickel layer [37], but also provide nucleation points for graphene deposition and enhance the adsorption performance of graphene. Xu et al. [38] prepared a Ni–graphene composite coating with brush-plating technology, and the composite coating had a better corrosion resistance as measured by electrochemical testing and immersion testing. Nie et al. [39] synthesized nickel nanoparticles/straight multi-walled carbon nanotubes (MWCNTs) nanohybrid using the in situ precipitation method, and prepared a sensor device for glucose detection. Due to the synergistic mechanism of nickel nanoparticles and MWCNTs, the sensor had excellent performance for glucose sensing.

Methane is a stable non-polar molecule, which is difficult to be adsorbed by conventional materials. Studies have found that cave fan is a caged supramolecular compound with a special encapsulation effect on methane molecules [40] which can adsorb methane molecules to achieve the purpose of detecting methane gas. Benounis [41] coated Poly catechol-styrene (PCS) fiber with Cryptophane-A and Cryptophane-E to create an alkane gas fiber sensor, which has better sensitivity, lower detection limit and stability. Boulart [42] reported on a low-cost sensor for measuring the concentration of dissolved methane. The sensor introduced Cryptophane-A into polydimethylsiloxane film to detect and quantify methane dissolved in aqueous media through refractive index modulation in situ. The limit of methane detection in the sensor was less than 0.2 nM, and the detection range was 1–300 nM. Chuanyi [43] designed a fiber-sensing element based on Cryptophane-A/silica nanowire luminescence quenching, which was used for dynamic monitoring of methane gas with concentrations below 3.5% and a detection lower limit of 0.1%. This sensor had fast response recovery, good repeatability, selectivity and stability. El-Ayle [44] also showed that Cryptophane has great potential, from synthetic development and binding properties to sensing applications, due to its special molecular recognition characteristics.

In this study, we combined graphene, nickel nanoparticles and Cryptophane materials with SAW technology, and deposited graphene–nickel–Cryptophane-A sensitive films on a SAW resonator with a central frequency of 204.3 MHz. A two-channel methane SAW sensor was developed and its performance was measured at room temperature.

## 2. Sensor Development

### 2.1. The Sensing Mechanism

The offset of the center frequency of the traditional acoustic surface gas sensor can be expressed as [45]:

(1) $$\frac{\Delta f}{f_{0}}=\frac{\Delta v}{v_{0}}=-c_{m}f_{0}\Delta \rho_{s}+c_{e}f_{0}h\Delta \left[\frac{4\mu /v_{0}^{2}\left(\eta +\mu \right)}{\eta +2\mu }\right]-\left[\frac{K^{2}}{2}\right]\Delta \left[\frac{\sigma_{s}^{2}}{\sigma_{s}^{2}+v_{0}^{2}C_{s}^{2}}\right]$$

where $f_{0}$ is the center frequency; $v_{0}$ is the undisturbed wave velocity; $c_{m}$ is the mass sensitivity coefficient; $\rho_{s}$ is the mass per unit area; h is the thickness of the film; $c_{e}$ is the elastic sensitivity coefficient of the substrate; $\sigma_{s}$ is the conductivity of the film surface; η is the volume of the film; μ is the shear modulus of the film; $K^{2}$ is the electromechanical coupling coefficient of the device and $C_{s}$ is the electrostatic capacity of the substrate electrode.

For ST-cut quartz crystal, when the sensitive film is an organic material, the viscoelastic effect of the gas to be measured on the sensitive film can be ignored due to the small shear modulus; that is, the second term on the right-hand side of Equation (1) can be ignored.

For traditional SAW gas sensors, the electrostatic capacity of the device is usually regarded as a constant value. When the sensitive film of the SAW gas sensor adsorbs the measured gas t, the surface conductivity ($\sigma_{s}$) of the sensitive film changes. The bound charges on the surface of the substrate will be rearranged by the excitation of conductive carriers in the sensitive film. As a result, the surface electric field intensity distribution changes, resulting in the change of gas SAW velocity and then the center frequency of the gas sensor shifts.

In this paper, a SAW gas sensor based on the coupling sensing mechanism of mass load effect and electric load effect is proposed. A layer of conductive graphene was deposited on the upper surface of the sensitive film, so that the adsorption of methane on the sensitive film will not change the conductivity ($\sigma_{s}$) of the film surface, or the $\sigma_{s}$ change is so small that it can be ignored. After $\sigma_{s}$ is fixed, the electrostatic capacity of the device should be taken as a variable affecting the center frequency of the sensor in order to couple the electrical load effect.

In this paper, a sensitive film was deposited on the upper surface of the cross-finger electrode of the sensor, and then a graphene layer was deposited on the upper surface of the sensitive film, so that the upper graphene and the cross-finger electrode constituted the upper and lower electrodes of the capacitance. In this way, the static capacitance of the SAW device can be changed from a constant value to a variable. Then, the value of $C_{s}$ was modified by adding the electrostatic capacity ($C_{s1}$) of the original device per electrode length and the new sensitive capacitance ($C_{s2}$). Then, formula 1 becomes:

(2) $$\frac{\Delta f}{f_{0}}=\frac{\Delta v}{v_{0}}=-c_{m}f_{0}\Delta \rho_{s}-\left[\frac{K^{2}}{2}\right]\Delta \left[\frac{\sigma_{s}^{2}}{\sigma_{s}^{2}+v_{0}^{2}C_{\left(C_{S1}+C_{S2}\right)}{}^{2}}\right]$$

**Figure 1:**
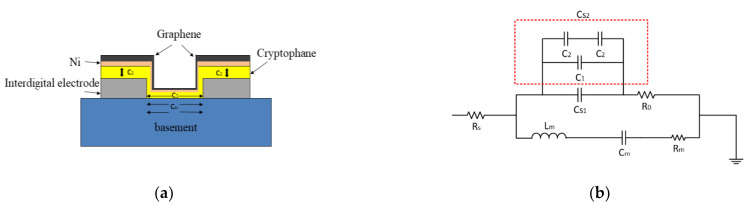
Equivalent circuit model: (**a**) Physical model; (**b**) Equivalent circuit.

As shown in Figure 1, on the basement of the SAW sensor, capacitor $C_{1}$ is constituted by the input cross-finger transducer electrode and the output cross-finger transducer electrode as the left and right plates of the capacitor in the horizontal direction. In the vertical direction to the substrate, the capacitor $C_{2}$ is composed of the input and output cross-finger transducer electrode and the upper graphene layer which is the second plate. Then, the new equivalent capacitance is:

(3) $$C_{S}=C_{S1}+C_{S2}$$

By Figure 1

(4) $$C_{S2}=C_{1}+1/2C_{2}$$

Formula (3) becomes:

(5) $$C_{S}=C_{S1}+C_{1}+1/2C_{2}$$

Since the width of the transducer of the SAW device is much larger than its thickness the value of $C_{1}$ is very small and negligible, so $C_{S}$ can be expressed as:

(6) $$C_{S}=C_{S1}+1/2C_{2}$$

Formula (2) becomes:

(7) $$\frac{\Delta f}{f_{0}}=\frac{\Delta v}{v_{0}}=-c_{m}f_{0}\Delta \rho_{s}-\left[\frac{K^{2}}{2}\right]\Delta \left[\frac{\sigma_{s}^{2}}{\sigma_{s}^{2}+v_{0}^{2}C_{\left(C_{S1}+\frac{1}{2}C_{2}\right)}{}^{2}}\right]$$

### 2.2. Design of Sensor Parts

**Figure 2:**
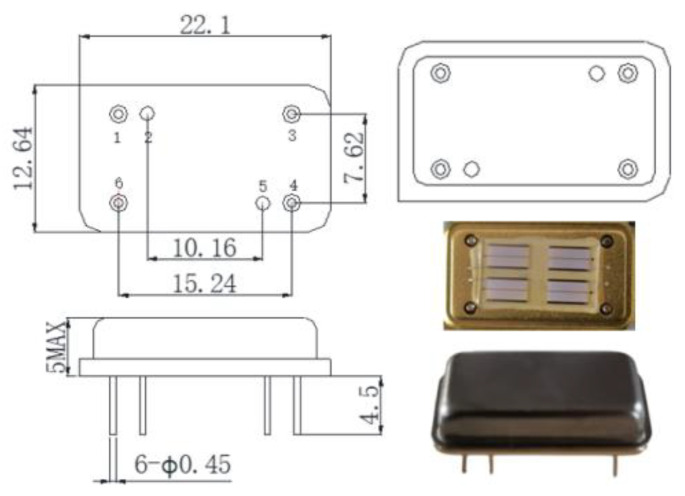
Device size and physical drawing.

The research group completed the design of sensor devices [46], and ST-X cut quartz—with a temperature coefficient of 0 and relatively low cost—was used as the substrate [47]. The size of the substrate material was 13 mm × 8 mm, and the thickness was 500 μm. The sensor adopted a delay-line aluminum fork electrode. SAW velocity of substrate material *V*_ST–x_ = 3158 m/s [48]. Center frequency $f_{0}$ = 204.3 MHz, acoustic aperture *W* = 2.5 mm, finger width dc = 3.86 μm, finger spacing $d_{0}$ = 3.86 μm. The specific size and physical model of the device are shown in Figure 2.

### 2.3. Preparation of Sensitive Film

**Figure 3:**
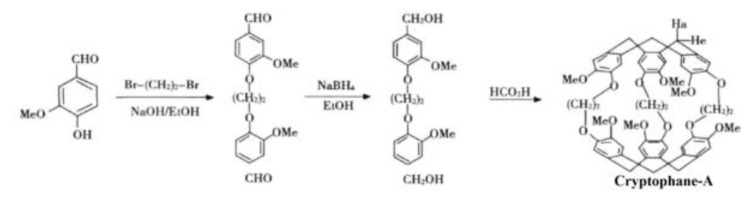
Cryptophane-A synthetic route.

Cryptophane-A was synthesized by a three-step method by using vanillin as the raw material, and the synthesis path is shown in Figure 3.

**Figure 4:**
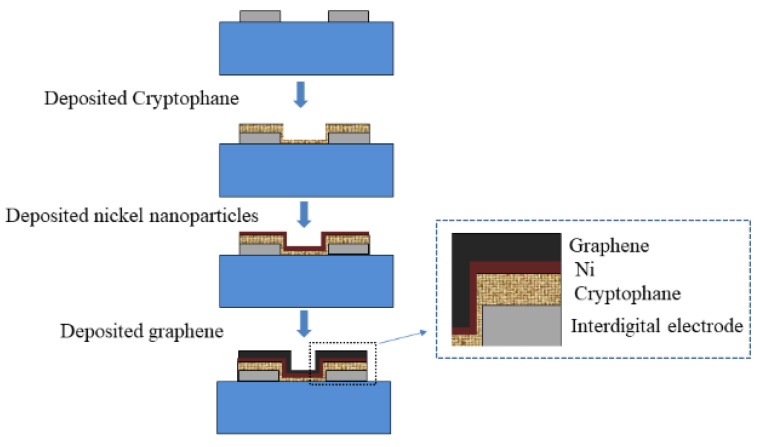
Sensitive film deposition process.

The graphene, nickel nanoparticles and Cryptophane-A were combined with SAW technology, and deposited the graphene–nickel–Cryptophane-A sensitive film on the SAW resonator with a central frequency of 204.3 MHz. The deposition process is shown in Figure 4.

**Figure 5:**
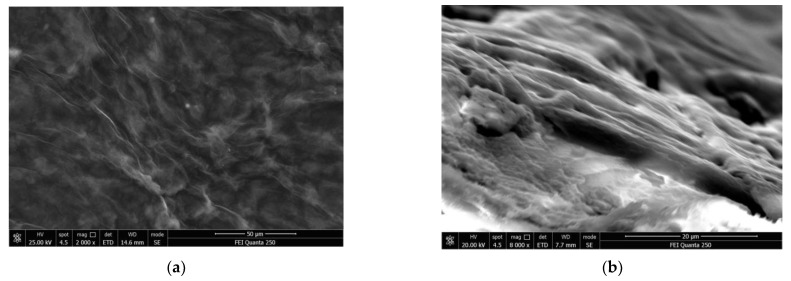
Film characterization: (**a**) Surface view; (**b**) Side view.

Firstly, 3.0 mg Cryptophane-A, 0.3 mg polyvinyl chloride and 0.6 mg dioctyl sebacate were dissolved in 2 mL tetrahydrofuran to prepare the sensitive film solution. The 0.3 mL solution was drip-coated onto the SAW device with a micro syringe, and then it was baked in an oven at 80 ℃ for 1 h. After the solvent was completely volatilized, Cryptophane-A film was obtained on the surface of the device. Ni nanoparticles were then electrodeposited by cyclic voltammetry, and graphene films were prepared by direct current electroplating to form the graphene–nickel–Cryptophane-A composite film. The film was characterized by an electron microscope of FEI Quanta 250 (Thermo Fisher Scientific Inc., Waltham, MA, USA) as shown in Figure 5. It can be seen that the surface of the film is flocculent and evenly distributed oxidized graphene, with a noticeable, layered structure on the side.

## 3. Results and Discussion

### 3.1. Performance Testing System of SAW Device

**Figure 6:**
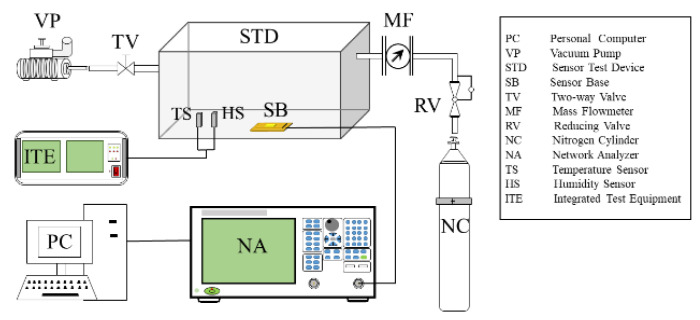
Performance testing system of SAW devices.

The SAW device is the main component of the SAW sensor and its characteristics will significantly affect the performance of the SAW sensor. The performance testing system of the SAW device is shown in Figure 6, including the computer (collecting signal of SAW device), vacuum pump, SAW device testing chamber, SAW device base, two-way valve, flow meter, pressure reducing valve, nitrogen cylinder (as protection gas), network analyzer, temperature sensor, humidity sensor and integrated test equipment.

On the premise of ensuring the air tightness of the SAW device-testing chamber, first turn on the integrated test equipment to make the temperature sensor and humidity sensor work normally. Then, open the valve door of the nitrogen cylinder and inject nitrogen. When the oxygen concentration tester shows the oxygen concentration is zero, close the two-way valve. Finally, the SAW device is tested by a network analyzer. The center frequency, insertion loss and other related data of the SAW device were recorded and analyzed.

### 3.2. Consistency Characteristics of Sensor Response

**Figure 7:**
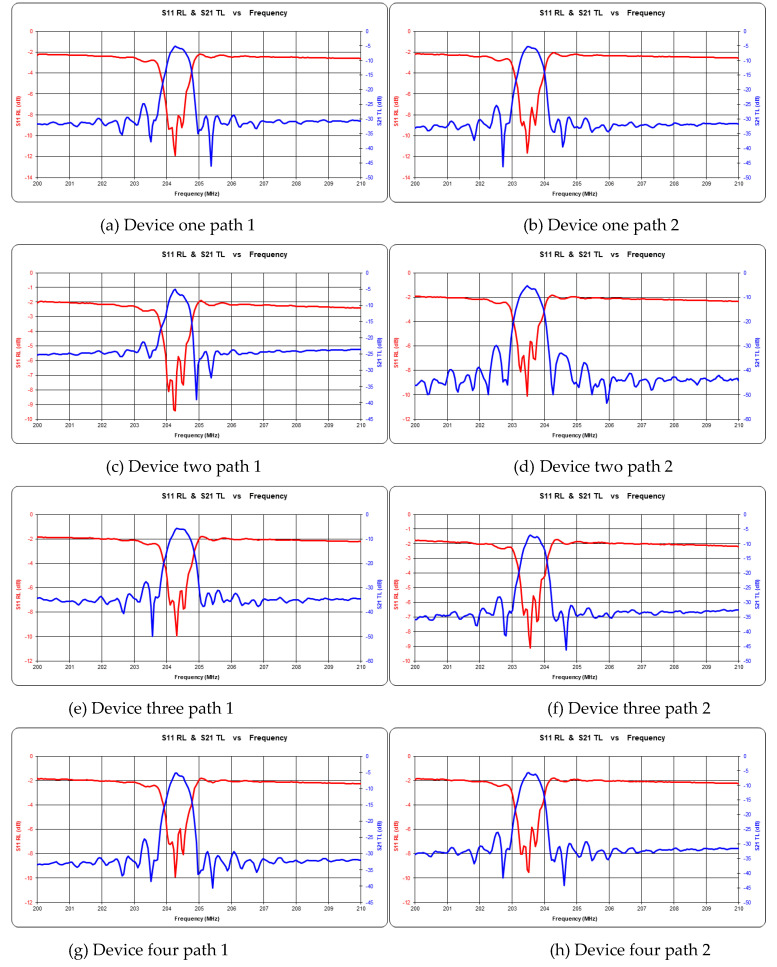
Conformance testing of SAW devices (The red line is the return loss, and the blue line is the transmission loss.).

The consistency of two signals of the delay linear sensor directly affects the performance of the sensor. The center frequency ($f_{0}$) of the typical delay-line acoustic meter device developed is about 204.3 MHz. The frequency test is shown in Figure 7, and the center frequency value is summarized in Table 1. The test range of the selected four components is between 204.26–204.31 MHz for path 1 and 203.46–203.56 MHz for path 2, which meet the design requirements. The difference of center frequency between two paths of each component is less than 0.8 MHz, which meets the requirement of surface acoustic-wave components.

### 3.3. Sensor Insertion Loss Test

Transmission loss, or insertion loss (IL), is an important parameter in SAW device design. The minimum insertion loss in the frequency band is regarded as the insertion loss of the device. In general, the insertion loss of SAW devices refers to working attenuation. The expression of insertion loss is:

(8) $$\mathrm{IL}=20\times \log_{10}\left|F\left\{V_{\mathrm{out}}\right\}/F\left\{V_{\mathrm{in}}\right\}\right|$$

**Figure 8:**
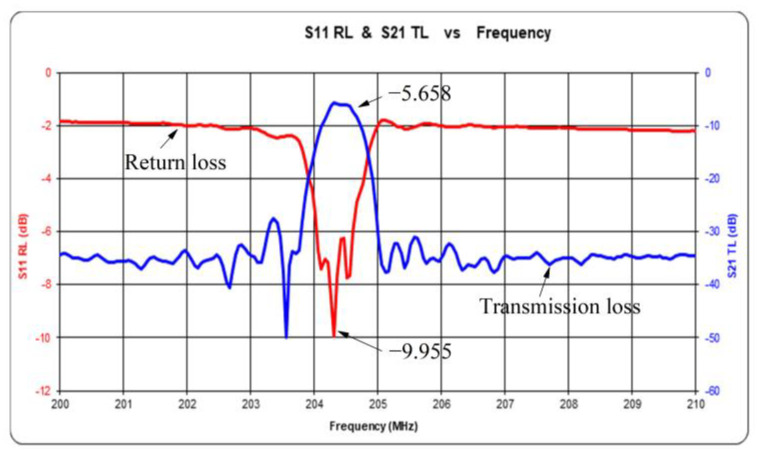
Return loss and transmission loss.

$F\left\{V_{\mathrm{in}}\right\}$ and $F\left\{V_{\mathrm{out}}\right\}$. are the Fourier transforms of the input and output voltages of the device, respectively. The result of time domain analysis is transformed into frequency domain by Fourier transform, and then the insertion loss of the device can be obtained by using formula (8). The test results are shown in Figure 8. The return loss and insertion loss are −9.955 dB and −5.658 dB, respectively.

### 3.4. Gas Sensing Test System

**Figure 9:**
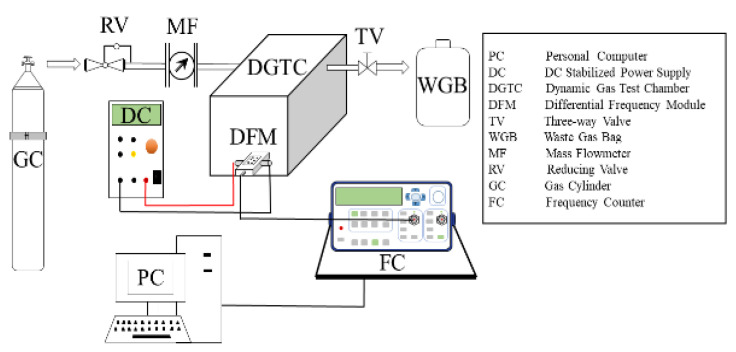
Gas-sensitive test system.

The gas sensitivity test system is shown in Figure 9. It consists of a personal computer (recording the frequency signal collected by the frequency meter), DC stabilized power supply (providing voltage), dynamic gas test-chamber (including sensors, differential frequency module and sensors for measuring methane concentration), three-way valves, gas cylinders (including pressure relief valves and mass flowmeters), frequency counter (collecting differential frequency signals) and waste gas bag (collecting exhaust gas).

The test process is as follows: first of all, check the air tightness of the entire pathway, and then turn on the DC-regulated power supply and frequency counter under the premise of ensuring no air leakage. Finally, methane gas was injected, and a personal computer was used to record and analyze the frequency value of the difference frequency signal.

### 3.5. Methane Response Characteristics of the Sensor

The relationship between the frequency offset (Δf) of the SAW sensor and the mass change (Δm) of its sensitive film is shown in Equation (9).

(9) $$\Delta f=f_{0}{}^{2}\left(k_{1}+k_{2}\right)\Delta m$$

where $f_{0}$ is the center frequency of the sensor, $k_{1}$, $k_{2}$ is the constant of piezoelectric substrate material.

**Figure 10:**
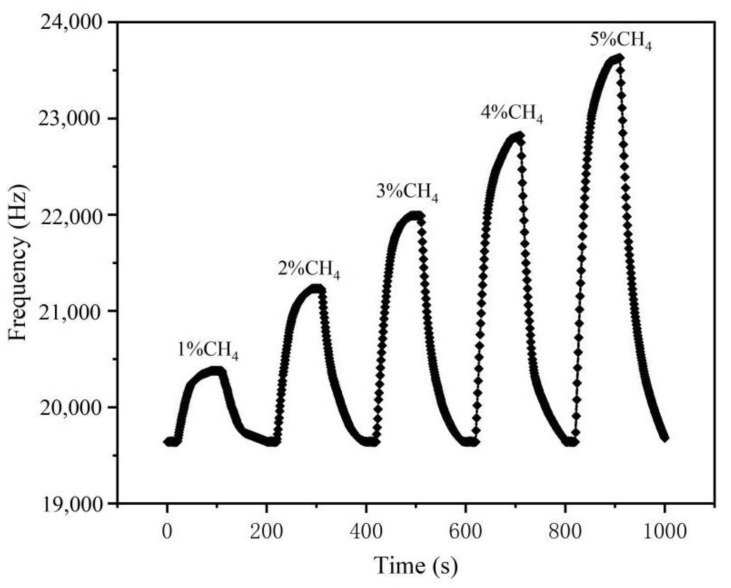
Frequency response recovery curve for 0–5% CH_4_.

The operating voltage of the acoustic surface sensor device is 12 V, the test temperature is 22 °C and the humidity is 46%. As can be seen in Figure 10, the difference frequency signal of the sensor showed good detection characteristics at methane concentrations of 0–5%.

**Figure 11:**
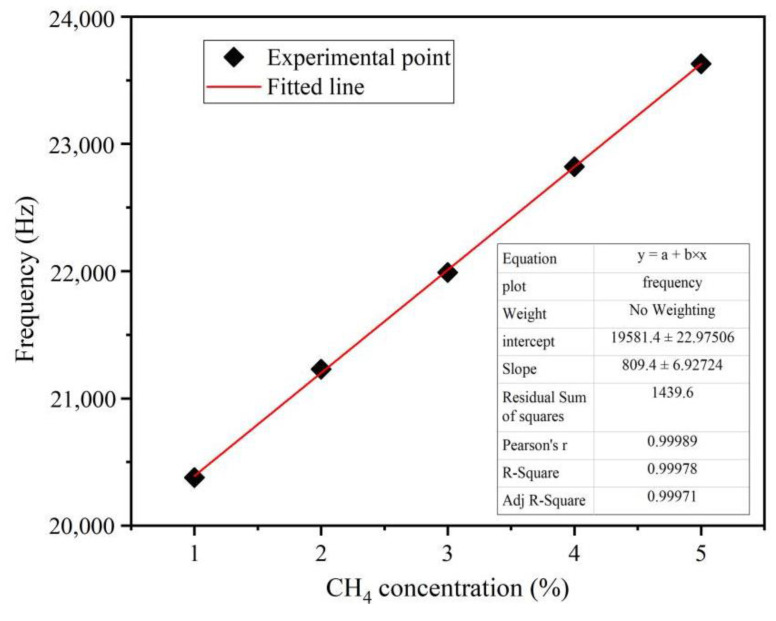
Linear fitting diagram of sensor response and CH_4_ concentration.

Figure 11 shows the sensitivity curve of the SAW sensor corresponding to methane gas concentration. As shown in Equation (9) above, the mass of the sensitive membrane will increase after the methane gas is adsorbed. Due to the mass loading effect, the frequency of the SAW sensor will also increase. 

The maximum response frequency values at 0–5% methane concentrations were extracted, respectively, and the response consistency at each concentration was observed and the linear fitting formula was obtained by linear fitting. The fitting situation is shown in Figure 11. The offset of the frequency of the SAW sensor is positively correlated with the change in methane concentration, and the fitting formula of the response consistency of the device was obtained.

According to the figure analysis, the test results of the device have a good concentration–frequency linear relationship for 0–5% concentration of methane, and the frequency response of the device is 809.4 Hz ± 6.93 Hz/(1% CH_4_).

The fitting formula of response consistency of the device is as follows:

(10) $$f_{freq}=19581.4+809.4\times C$$

where $f_{freq}$ is the maximum response frequency, C is the CH_4_ concentration (0–5%).

### 3.6. Characteristics of the Sensor Response Time

**Figure 12:**
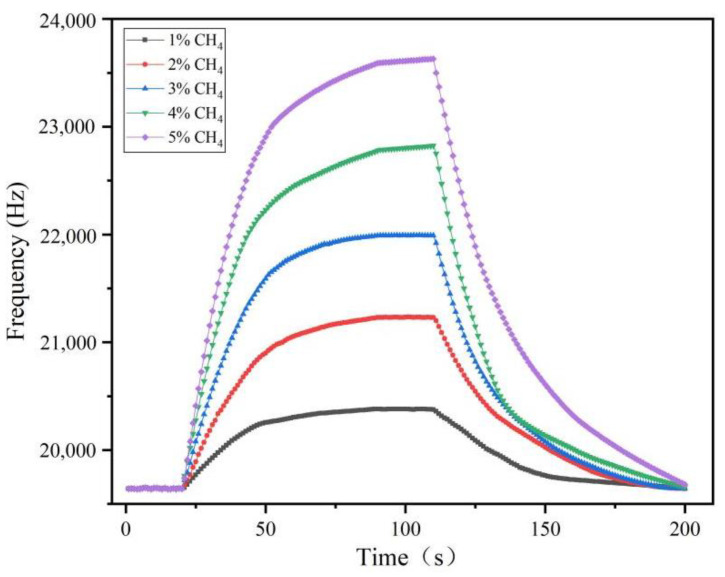
Methane sensor response–recovery characteristic curve.

**Figure 13:**
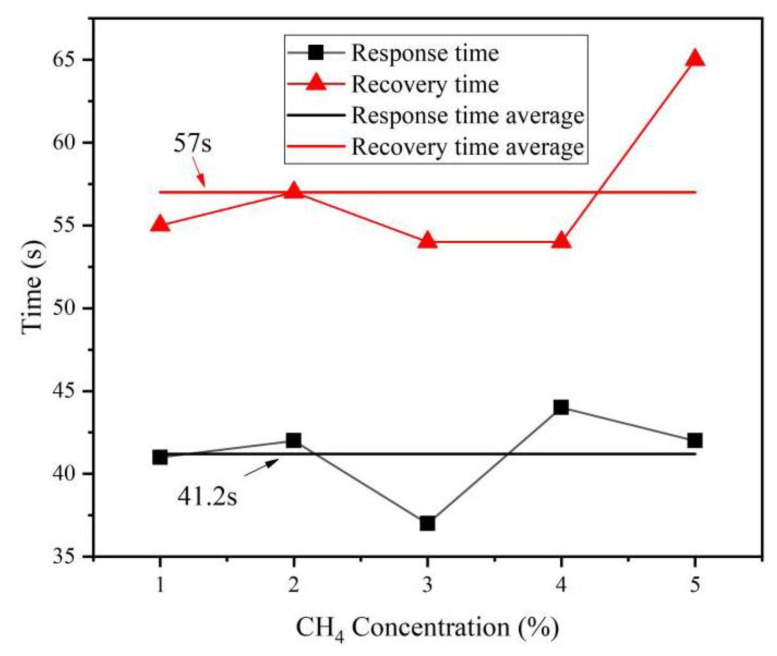
Methane sensor response–recovery time curve.

Under the test temperature of 22 °C and humidity of 46%, the sensor operating voltage was set to 12 V, and the response recovery curves of methane concentration from 1% to 5% were measured by controlling the concentration of the incoming methane gas, as shown in Figure 12. The results showed that the sensor using the graphene–nickel–Cryptophane-A composite sensitive membrane had a good response and recovery performance for different methane concentrations. Ninety percent of the response and recovery times fluctuated slightly, with an average response time of 41.2 s and a recovery time of 57 s, as shown in Figure 13. The response speed is faster than the recovery speed, indicating that the absorption of composite sensitive membrane film is better than desorption.

### 3.7. Characteristics of the Sensor Selection

**Figure 14:**
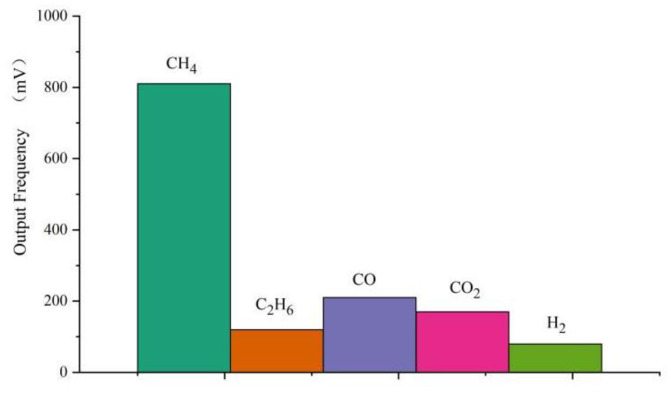
Sensor selectivity experiment.

In addition to methane, coal mine gas often contains background gases such as CO, CO_2_, C_2_H_6_ and H_2_. It is necessary to consider the response sensitivity of the sensor to the background gases. Under the test temperature of 22 °C, the humidity of 46% and sensor power supply of 12 V, the sensitivity of 1 wt.% MWCNTs modified sensor to four common interfering gases at 1% CH_4_ volume concentration was measured, as shown in Figure 14. It can be seen that the sensor has the highest sensitivity to methane, 810 Hz/1% CH_4_; the sensitivity of ethane is about 120 Hz/1% C_2_H_6_; the sensitivity of CO is about 200 Hz/1% CO; the sensitivity of CO_2_ is about 170 Hz/1% CO_2_; Hydrogen has the lowest sensitivity, only 80 Hz/1% H_2_. Experiments show that the selectivity of the sensor for methane is significantly better than that of the other four gases.

### 3.8. Humidity Influence on the Response of the Sensor

**Figure 15:**
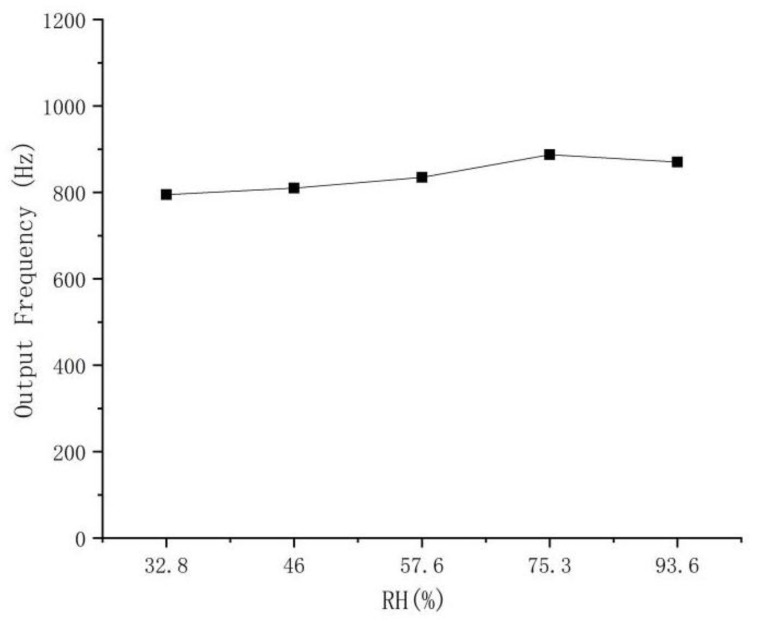
Experiment of humidity influence.

An underground coal mine is an environment where humidity changes, with the maximum humidity exceeding 90%, sometimes reaching 100%. Here, we measured the sensor output frequency values, at 32.8%, 46%, 57.6%, 75.3%, and 93.6% humidity, at 22 ℃ ambient temperature, as shown in Figure 15. With the increase in humidity, the output frequency of the sensor shows a trend of rising first and then decreasing. At 75.3% RH, the output frequency increases by 92 Hz compared with 32.8% RH. Compared with the sensitivity of the sensor response, it is equivalent to the output offset of 0.1% CH_4_. The experiment shows that the humidity has a certain influence on the output of the sensor, but the overall influence is still within the required range of measurement accuracy (0.1%).

## 4. Conclusions

In this study, 0–5% concentration of methane gas was taken as the detection target under the test temperature of 22 ℃ and humidity of 46%. By depositing the graphene–nickel–Cryptophane-A composite sensitive film, we designed an acoustic surface wave methane sensor coupled with mass load effect, and obtained the following conclusions: The center frequency of sensor consistency is about 204.3 MHz. The return loss and insertion loss are −9.955 dB and −5.658 dB, respectively. The SAW methane sensor was prepared by the “three-step method” and was combined with graphene and nickel nanoparticles. The graphene–nickel–Cryptophane-A composite sensitive film was deposited on the surface acoustic wave device. In the range of 0–5% methane concentration, the response frequency of the methane sensor based on graphene–nickel– Cryptophane-A composite sensitive film has a good linear relationship with methane concentration, and the average response sensitivity is 809.4 Hz ± 6.93 Hz/(1% CH_4_). The average response time of 90% was 41.2 s, and the average recovery time of 90% was 57 s. Meanwhile, the sensor has good selectivity for methane and is less affected by humidity.

## Figures and Tables

**Table 1:** Sensor conformance test center frequency table.

Device	Path 1 (MHz)	Path 2 (MHz)
1	204.26	203.46
2	204.26	203.46
3	204.31	203.56
4	204.26	203.51

## Data Availability

The data presented in this study are available on reasonable request from the corresponding author.
